# Radiation exposure from fluoroscopy during fixation of hip fracture and fracture of ankle: Effect of surgical experience

**DOI:** 10.4103/0019-5413.43398

**Published:** 2008

**Authors:** Rajesh Botchu, Kassetti Ravikumar

**Affiliations:** Department of Orthopaedics, Maidstone Hospital, Maidstone, UK

**Keywords:** Experience, fixation, fracture, radiation, surgeon

## Abstract

**Background:**

Over the years, there has been a tremendous increase in the use of fluoroscopy in orthopaedics. The risk of contracting cancer is significantly higher for an orthopedic surgeon. Hip and spine surgeries account for 99% of the total radiation dose. The amount of radiation to patients and operating surgeon depends on the position of the patient and the type of protection used during the surgery. A retrospective study to assess the influence of the radiation exposure of the operating surgeon during fluoroscopically assisted fixation of fractures of neck of femur (dynamic hip screw) and ankle (Weber B) was performed at a district general hospital in the United Kingdom.

**Materials and Methods:**

Sixty patients with undisplaced intertrochanteric fracture were included in the hip group, and 60 patients with isolated fracture of lateral malleolus without communition were included in the ankle group. The hip and ankle groups were further divided into subgroups of 20 patients each depending on the operative experience of the operating surgeon. All patients had fluoroscopically assisted fixation of fracture by the same approach and technique. The radiation dose and screening time of each group were recorded and analyzed.

**Results:**

The radiation dose and screening time during fluoroscopically assisted fixation of fracture neck of femur were significantly high with surgeons and trainees with less than 3 years of surgical experience in comparison with surgeons with more than 10 years of experience. The radiation dose and screening time during fluoroscopically assisted fixation of Weber B fracture of ankle were relatively independent of operating surgeon's surgical experience.

**Conclusion:**

The experience of operating surgeon is one of the important factors affecting screening time and radiation dose during fluoroscopically assisted fixation of fracture neck of femur. The use of snapshot pulsed fluoroscopy and involvement of senior surgeons could significantly reduce the radiation dose and screening time.

## INTRODUCTION

The use of fluoroscopy has increased tremendously in field of orthopedics.^1^,^2^ The risk of an orthopedic surgeon contracting cancer is significantly higher than that of a nonorthopedic professional and eight times more than that of an unexposed worker.^3^ The hip and spine surgeries account for most of the total radiation exposure.^1^ The amount of radiation exposed to individual surgeons is influenced by many factors.^4^,^5^ These include the type and difficulty of the surgical procedure, patient's position, and radiation protection measures used. The role of experience of the surgeon involved in the radiation exposure has not been studied well.^3^,^4^,^6^

The aim of the study was to assess the effect of surgical experience on the radiation exposure and screening time during fluoroscopically assisted fixation of undisplaced fractures of neck of femur (dynamic hip screw) and ankle (Weber B).

## MATERIALS AND METHODS

A retrospective study was conducted at a district general hospital in the United Kingdom from 2003 to 2005. The surgeons were divided into three different groups based on their years of operative experience [Table 1]. Surgeons and trainees with operative experience of less than 3 years were grouped together in Group 1; surgeons and trainees with surgical experience between 3 and 10 years were put in Group 2, and surgeons with more than 10 years were in Group 3.

**Table 1:** Surgical experience

Group I	< 3 years
Group II	3–10 years
Group III	> 10 years

A total of 700 patients with fixation of fracture of neck of femur underwent fluoroscopically assisted fixation of hip (dynamic hip screws), and 225 patients with ankle fracture underwent fluoroscopically assisted fixation during 2003–2005. Only patients with undisplaced intertrochanteric fracture were included in the hip group. Patients with multiple fractures, communited fracture, compound fracture, and significant morbidity were excluded.

Patients with a fracture of lateral malleolus without communition was included in the ankle group. Patients with polytrauma, compound fracture, medial malleolar fracture, and communition were excluded.

In each subgroup of the hip group, 20 consecutive patients who satisfied the inclusion criteria and were operated by the appropriate surgeon were included (20 patients operated by Group 1, 20 patients operated by Group 2, and 20 patients operated by Group 3). The age and sex of the patients were comparable in the hip and ankle group.

Similarly in the ankle group, in each subgroup, 20 consecutive patients who satisfied the inclusion criteria and were operated by the appropriate surgeon were included (20 patients operated by Group 1, 20 patients operated by Group 2, and 20 patients operated by Group 3).

All screening was performed using a mobile C arm fluoroscopy unit (Philips Libra, Philips Medical Systems, Best, The Netherlands). The total filtration of the x-ray tube was 6.35 mm; the focus to detector distance was 99.5 cm, and the diameter of the routinely used input field of view was 17 cm. The dose of the patient was routinely monitored and recorded in accordance with the requirements of Regulation 7 of The Ionising Radiation (Medical Exposure) Regulations [IR(ME)R] 2000 by means of a dose area product (DAP) meter permanently built into the x-ray tube housing.^7^

DAP is the currently accepted method of assessing the radiation exposure in complex diagnostic x-ray procedures and is measured in Gy cm^2^. The DAP meter was calibrated following the procedure described in the National Protocol for Patient Dose Measurement in Diagnostic Radiology.^8^

In addition to the DAP value, the overall fluoroscopic screening time (minutes) was recorded for each patient.

All the patients with fracture neck of femur underwent dynamic hip screw fixation by lateral approach. The patients with Weber B fracture of ankle underwent fixation of lateral malleolus using a one-third tubular plate using a lateral approach.

## RESULTS

The radiation dose and screening times of the various groups were compared using Student *t* test, and *P* values were calculated.

### Fixation of fracture of neck of femur

The radiation dose (DAP) and screening time during fixation of a hip fracture were almost three times more with surgeons and trainees with less than 3 years of operative experience (Group I) when compared with that of the surgeons with more than 10 years of surgical experience (Group III) [Table 2] (*P* = 0.0005).

**Table 2:** Radiation dose and screening time for fixation of Tronzo 2 fracture neck of femur

	Screening time (min)		Dose area product (Gy cm^2^)	
		
	Average	Median	Average	Median
Group I	1.1882	1.23	1.6215	1.4
Group II	0.6657	0.57	1.111	1.18
Group III	0.492	0.36	0.5932	0.56

### Fixation of fracture of ankle

The radiation dose and screening time during fixation of ankle were not significantly affected by surgical experience of the operating surgeon, and they were comparable among all the three groups [Table 3] (*P* = 0.1015).

**Table 3:** Radiation dose and screening time for fixation of Weber B fracture of ankle

	Screening time (min)		Dose area product (Gy cm^2^)	
		
	Average	Median	Average	Median
Group I	0.21	0.21	0.1933	0.19
Group II	0.2095	0.23	0.1518	0.144
Group III	0.1518	0.145	0.1237	0.119

## DISCUSSION

The image intensifier can be considered as the backbone for intraoperative fracture fixation.^2^,^5^ Mastrangelo *et al*. had shown a significant increase in the incidence of cancers in orthopedic workers.^6^ The incidence was four times higher in an orthopedic surgeon than in a nonorthopedic professional and eight times more than that in an unexposed worker. He also emphasized the significance of radiation protection measures and warned against complacency.

Radiation exposure can be decreased significantly by radiation protection measures and by increasing the distance between the source of radiation and the subject.^1^,^2^ Mehlman and DiPasquale had demonstrated that working more than 36 inches from the beam significantly decreases the radiation dose.^9^ Giannoudis *et al*. had demonstrated a significant correlation between the radiation dose administered and experience of the radiographer.^3^ The radiation dose of all the subjects included in the study conducted by Smith *et al*. was below the recommended limits.^10^

Crawley and Rogers in their study found the median radiation dose for dynamic hip screw fixation and ORIF ankle to be 2.58 Gy cm^2^ and 0.39 Gy cm^2^, respectively, whereas the median screening time was 0.90 and 0.55 min.^1^

**Figure 1:**
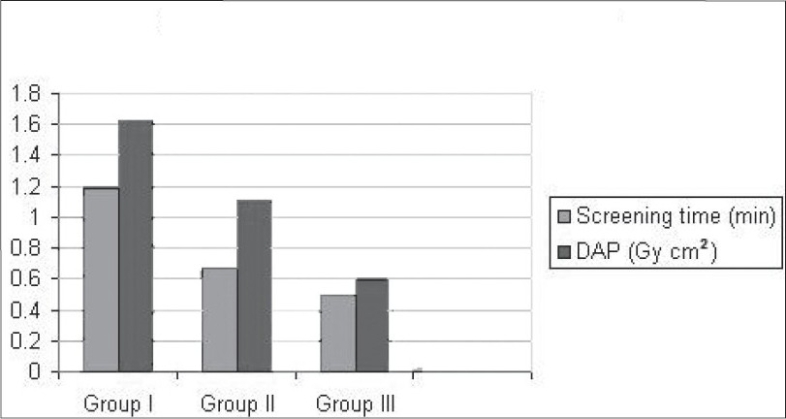
Screening times and radiation dose in fixation of fracture neck of femur

**Figure 2:**
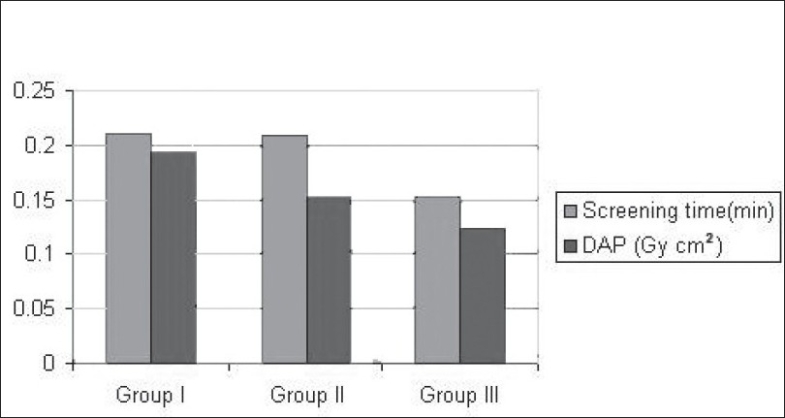
Screening times and radiation dose in fixation of ankle fracture (Weber B)

In this study, overall patient doses as monitored by DAP were reassuringly well below these published values. The radiation exposure during fixation of fractures of hip was significantly higher when they were performed by surgeons with fewer than 3 years of operative experience, and this was statistically significant (*P* = 0.0005 ) [Figure 1]. In patients who underwent fixation for fractures of ankle, the radiation exposure and screening time was not significantly dependent on the surgical experience of the operating surgeon (*P* = 0.1015) [Figure 2].

## CONCLUSIONS

This study shows that the experience and training of the operating surgeon is one of the most important factors determining the radiation exposure to patients in fixation of hip fracture. The involvement of senior surgeons in hip fracture fixation is likely to reduce the radiation dose to patients and surgeons. We endorse the practice of using snap shot pulsed fluoroscopy, last image hold, and good set up geometry as a means of dose optimization. The practice of continuous screening during fixation of fractures of neck of femur and ankle must be discouraged.
